# Knee osteoarthritis accelerates amyloid beta deposition and neurodegeneration in a mouse model of Alzheimer’s disease

**DOI:** 10.1186/s13041-022-00986-9

**Published:** 2023-01-02

**Authors:** Deepak Prasad Gupta, Young-Sun Lee, Youngshik Choe, Kun-Tae Kim, Gyun Jee Song, Sun-Chul Hwang

**Affiliations:** 1 Department of Medical Science, College of Medicine, Catholic Kwandong University, Gangneung, Gangwon-Do Republic of Korea; 2 Translational Brain Research Center, International St. Mary’s Hospital, Catholic Kwandong University, Incheon, Republic of Korea; 3 Korea Brain Research Institute, Daegu, Republic of Korea; 4 Department of Orthopaedic Surgery, Gyeongsang National University College of Medicine, Gyeongsang National University Hospital, Jinju-Si, Gyeongsangnam-Do Republic of Korea

**Keywords:** Knee osteoarthritis, Amyloid deposition, Neurodegeneration, Alzheimer’s disease, Neuroinflammation

## Abstract

Knee osteoarthritis (OA) is characterized by knee cartilage degeneration and secondary bone hyperplasia, resulting in pain, stiffness, and gait disturbance. The relationship between knee OA and neurodegenerative diseases is still unclear. This study used an Alzheimer’s disease (AD) mouse model to observe whether osteoarthritis accelerates dementia progression by analyzing brain histology and neuroinflammation. Knee OA was induced by destabilizing the medial meniscus (DMM) in control (WT) and AD (5xFAD) mice before pathological symptoms. Mouse knee joints were scanned with a micro-CT scanner. A sham operation was used as control. Motor and cognitive abilities were tested after OA induction. Neurodegeneration, β-amyloid plaque formation, and neuroinflammation were analyzed by immunostaining, Western blotting, and RT-PCR in brain tissues. Compared with sham controls, OA in AD mice increased inflammatory cytokine levels in brain tissues. Furthermore, OA significantly increased β-amyloid deposition and neuronal loss in AD mice compared to sham controls. In conclusion, knee OA accelerated amyloid plaque deposition and neurodegeneration in AD-OA mice, suggesting that OA is a risk factor for AD.

## Introduction

Together with the increasing average human lifespan, the number of people suffering from geriatric diseases is augmenting. This requires increasing research on the common pathogenesis of osteoarthritis (OA) and Alzheimer’s disease (AD), which are major geriatric diseases. Recent studies have reported that the mechanism of chronic inflammation is closely related to the onset of degenerative diseases such as AD and OA [1]. Knee OA is the most common joint disease, affecting 10% of men and 13% of women over the age of 60 [2]. According to Xue, in 2018, the prevalence of dementia in an OA group was higher than that in the non-OA group (HR 1.42, 95% CI, 1.30–1.54); OA treatment reduced the risk of dementia [3]. Despite ongoing epidemiological studies on the correlation between dementia and OA [4–6], there is still no direct evidence showing its causal relationship.

AD, responsible for the largest proportion of dementia, is a chronic neurodegenerative disease with various known risk factors, but its causes and specific treatment remain unknown. Current AD medications, including acetylcholinesterase inhibitors and memantine (an NMDA receptor antagonist), improve cognitive decline only temporarily, and cannot stop or reverse the progression of dementia. Specific strategies are needed to overcome the high failure rate in developing new drugs for AD [7].

Neuroinflammation, which is the major pathophysiology of AD, leads to neurodegenerative processes including activation of microglia and astrocytes, release of inflammatory substances (e.g., nitric oxide (NO), cytokines, and chemokines), dysfunction of microglial clearance capacity of amyloid plaques, accumulation of amyloid plaques, and neuronal cell death [8]. Inflammatory activation of microglia is considered a pathological marker and an important mechanism of degenerative brain disease progression. Recently, growing evidence has indicated the impact of systemic inflammation on neuroinflammation [9, 10]. Despite the existence of the blood–brain barrier (BBB), it is not clear how peripheral inflammatory diseases affect neuroinflammation.

Few studies have demonstrated common molecular mechanisms and a direct causal relationship between AD and OA. Therefore, it is required to identify the causal relationship between AD and OA and to find a therapeutic target through related pathological mechanisms. In this study, we identified a causal relationship, suggesting that OA could be a risk factor for AD using mouse animal models.

## Materials and methods

### Animals

All animal experiments were performed according to the approved guidelines by the Catholic Kwandong University animal committee, International Saint Mary’s Hospital (No. CKU 2020-012). 5xFAD transgenic mice (B6SJL-Tg (APPSwF1Lon, PSEN1 × M146L × L286V) 6799Vas/Mmjax) were purchased from the Jackson Laboratory (Bar Harbor, ME, USA) and inbred by crossing with C57BL/6 mice. All mice were genotyped using polymerase chain reaction (PCR) from genomic DNA extracted from the mouse tail biopsy. Animals were housed with 24-h food and water with 12-h dark/light cycles at a constant temperature of 23 °C.

### Surgical destabilization of the medial meniscus (DMM)

OA was induced by DMM as described previously [11, 12]. Animals were anesthetized under 3% isoflurane, and the right knee joint was exposed through a medial parapatellar approach, making a 1-cm longitudinal medial parapatellar incision. The medial meniscus, located between the medial condyle of the femur and the medial plateau of the tibia, was identified and severed using a No. 10 surgical blade. The surgeries were either a sham, performed by just opening the joint capsule and DMM, in which the menisco-tibial ligament was cut to destabilize the medial meniscus. The knee was flushed with saline, and the joint capsule incision was closed with a No. 6 absorbable suture.

### Y-maze test

A Y-maze test was performed to measure short-term spatial memory in mice. Mice were habituated to the testing room for 30 min. A mouse was placed in an arm of the Y-maze and allowed to freely explore the arms for 10 min. Spontaneous alterations were recorded and analyzed by a tracking software (EthoVision XT, Noldus, Wageningen, the Netherlands) connected to a video recording camera. The spontaneous alteration was calculated as a percentage by dividing the number of alterations by the total number of alterations.

### Limb harvesting and micro-CT scanning

Following behavioral tests, both hind limbs were dissected immediately and fixed in 4% paraformaldehyde for 24 h after skin and extra tissue removal. Mouse hind limbs were scanned using a desktop micro-CT (SkyScan 1176, Bruker, MicroCT, Kontich, Belgium). The organs were scanned at 35–60 kV, 200 µA, 0.5 mm aluminum filter, 360° rotation, and a voxel size range of 1.65–6.56 µm. Micro-CT projections were back-reconstructed using the NRecon software (NReconServer64bit, Bruker, MicroCT, Kontich, Belgium) and volume-rendered and visualized in 3D with the CTVox software (Bruker, MicroCT, Kontich, Belgium).

### Immunofluorescence staining of brain tissues

Mice were anesthetized and perfused intracardially with 0.9% saline. Knee and brain samples were collected and post-fixed for 12 h in 4% paraformaldehyde. Brain tissue sections of 20 μm thickness were acquired with a CM3050S microtome (Leica, Wetzlar, Germany). Brain tissue sections were permeabilized in 0.3% TritonX-100 containing phosphate-buffered saline (PBST), followed by blocking with 0.3% normal donkey serum and 1% bovine serum albumin for 60 min at room temperature. The sections were then incubated with ionized calcium binding adaptor molecule 1 (Iba1, microglia marker, 1:200, goat, Novus Biologicals, NB100-1028), β-amyloid (amyloid plaques marker, 1:200, mouse, Santa Cruz, sc-28365), or Neu-N (neuron marker, 1:1000, rabbit, Millipore, ABN78) as described previously [13]. Sections were mounted with DAPI (Molecular Probes, Life Technologies).

### Microscopy and quantification

Fluorescent signals were captured with a Carl Zeiss Axio Imager Z1 fluorescence microscope equipped with ApoTome (Carl Zeiss MicroImaging, Inc.) using the same exposure time for each image. Images were analyzed using ImageJ software (NIH), converted to grayscale, and used the same post-acquisition threshold for analysis. Manual counting of Iba1^+^ cells or amyloid plaques was performed with ImageJ software, using the ‘analyze’ grid, by analyzers blind to mouse genotype and treatment groups. Five to 10 images from one brain slice were analyzed for quantification of the number and area (μm^2^) of Iba1 cells and amyloid plaques.

### Quantitative RT-PCR analysis

Total RNA was extracted from mouse brain frontal cortex tissue by using TRIZOL (Invitrogen, Carlsbad, CA, USA). Total RNA (2 μg) was used to synthesize cDNA using the Superscript II reverse transcriptase (Invitrogen) and an oligo (dT) primer. Conventional PCR was performed with the specific primer sets in Table 1 using a T100 Thermal Cycler (Bio-Rad, Richmond, CA, USA). PCR products were electrophoresed on a 2% agarose gel. PCR bands were imaged under ultraviolet light and quantified by ImageJ. Quantitative real-time PCR experiments were carried out using the One Step SYBR PrimeScript RT-PCR Kit (Takara Bio, Otsu, Shiga, Japan), and the ABI Prism 7000 Sequence Detection System (Applied Biosystems, California, CA, USA). As an internal reference gene, glyceraldehyde 3-phosphate dehydrogenase (GAPDH) or cyclo were used. All experiments were performed at least in triplicate, and relative expression was calculated using the comparative threshold cycle method.

**Table 1:** PCR conditions and sequence information for the primers

Target genes	Forward primer (5′ → 3′)	Reverse primer (5′ → 3′)	Temp (°C)	Cycles
TNF-α	CATCTTCTCAAAATTCGAGTGACAA	ACTTGGGCAGATTGACCTCAG	60	25
iNOS	CCCTTCCGAAGTTTCTGGCAGCAGC	GGCTGTCAGAGCCTCGTGGCTTTGG	70	30
IL-1β	CTTTGAAGAAGAGCCCATCC	TTTGTCGTTGCTTGGTTCTC	Realtime	40
IL-6	CCAATTTCCAATGCTCTCCT	ACCACAGTGAGGAATGTCCA	Realtime	40
Cyclo	TGGAGAGCACCAAGACAGACA	TGCCGGAGTCGACAATGAT	Realtime	40
GAPDH	ACCACAGTCCATGCCATCAC	TCCACCACCCTGTTGCTGTA	60	25

### Western blot analysis

Proteins were extracted with RIPA lysis buffer (50 mM Tris–HCl, 150 mM NaCl, 0.02% sodium azide, 0.1% SDS, and 1% NP-40). Equal amounts of protein were resolved on a 10% SDS–polyacrylamide gel and transferred to polyvinylidene difluoride membranes (Bio-Rad Laboratories, California, USA). The blots were blocked with 4% skim milk in PBS with 0.1% Tween-20 and then incubated with primary antibodies, GFAP (1:1000, Rabbit, DAKO, Z0334), MBP (1:1000, Rabbit, Sigma, M3821), and GAPDH (1:2000, Rabbit, Cell Signaling, #2118) overnight at 4 °C. Specific bands for the primary antibodies were detected with horseradish peroxidase-conjugated secondary antibodies (1:2000) using an ECL compound solution (SuperSignal West Femto; Thermo Fisher, Franklin, MA, USA).

### Statistical analysis

Statistical analyses were performed using one-way analysis of variance (ANOVA) followed by Tukey’s multiple comparison test or unpaired t-tests, assuming equal variances were used to compare the groups of interest. Results are presented as the mean ± standard error of the mean. A p-value < 0.05 was considered significant for all tests. All analyses were carried out using GraphPad Prism version 8.00 (GraphPad Software, San Diego, CA, USA).

## Results

### OA did not induce neuroinflammation and amyloid plaque deposition in wild-type control mice

**Fig. 1:**
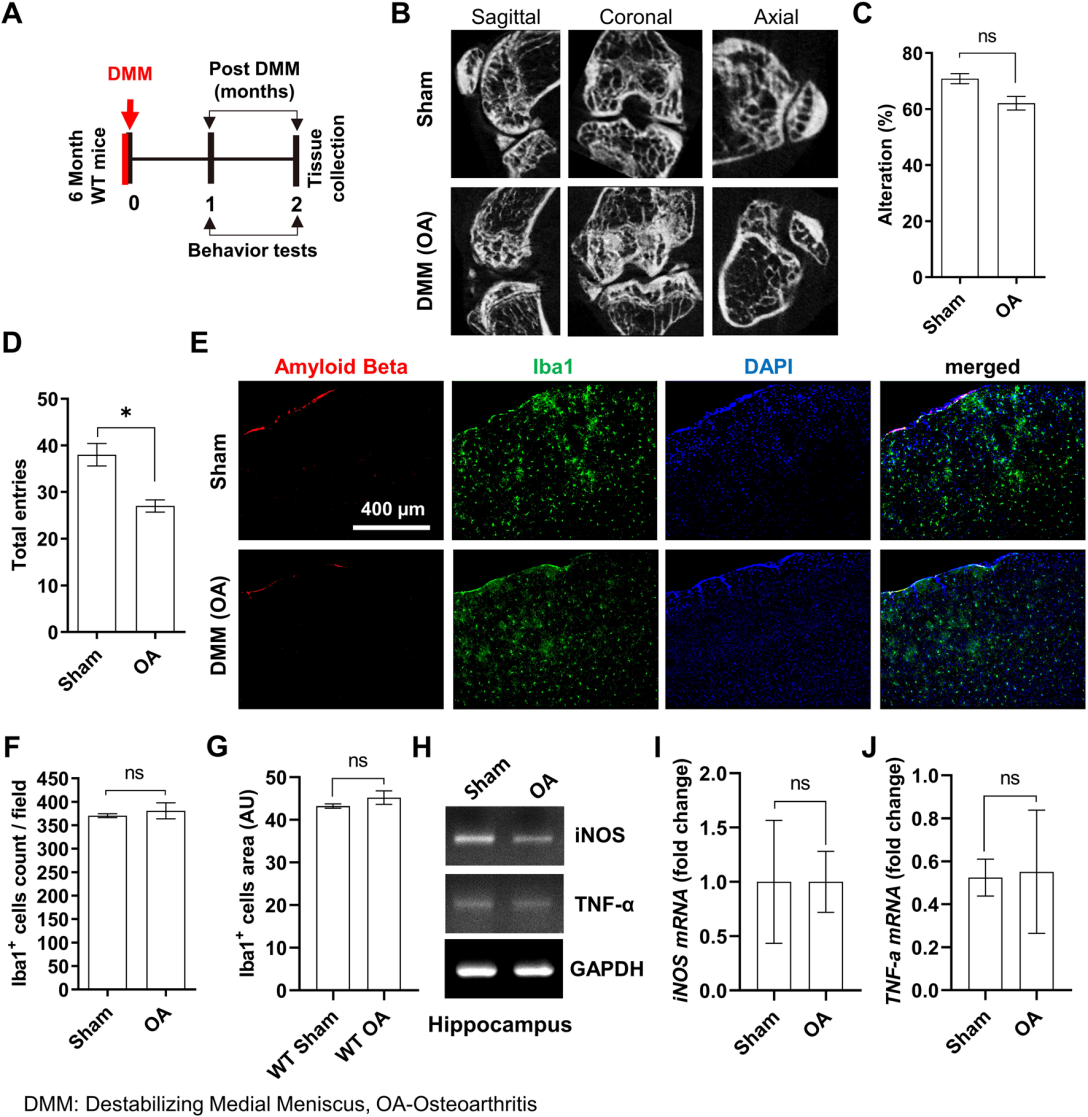
Osteoarthritis (OA) induction by surgical DMM did not induce AD pathology in WT mice. **A** Timeline of surgical DMM and tissue collection in 6-month-old WT mice. **B** Representative images showing the sagittal, coronal, and axial views from the micro-CT scan of the subchondral bone acquired 2 months after surgical DMM. Spatial working memory tests in WT and TG mice. **C** Percentage of Y-maze alterations. **D** Total number of arm entries (n = 5 for the sham group, n = 10 for the OA group). **E** Representative images showing Iba-1 immunofluorescence staining in sham and surgical DMM-induced mouse brain tissues from WT mice. **F** Iba-1-positive cell count. **G** Area of Iba-1-positive cells in the cortical brain region (5 images/brain, n = 4). **H** *iNOS* and *TNF-α* mRNA expression in the hippocampus. Relative mRNA expression for iNOS (**I**) and TNF-α (**J**), respectively (n = 4/group). The band intensities of iNOS and TNF-α were normalized to GAPDH. Values are presented as mean ± SEM. Student’s *t*-test was used for statistical analyses. *P < 0.05 was considered statistically significant for the comparison between sham control and OA mice; ns, not significant

Patients with OA typically represent progressive destruction of the extracellular matrix of articular cartilage, bone remodeling, and synovial inflammation. To induce human-like OA in mice, surgical DMM was performed in middle-aged wild-type (WT) control mice (6 months old); histological knee evaluations were performed 2 months after surgery (Fig. 1A). Micro-CT scan images of the knee showed severe bone remodeling in the OA group compared with animals with sham surgery (Fig. 1B). Y-maze tests were performed 2 months after DMM surgery to evaluate cognitive behaviors in mice with induced OA. Although the number of total entries was significantly lower in the OA group, the alternation rate in the Y-maze did not differ between the groups of mice, suggesting that OA did not induce cognitive deficits in WT control mice (Fig. 1C and D). Next, neuroinflammation in the brain was examined by immunostaining using the microglial marker Iba-1. In WT mice, OA induction did not trigger microglial hyperactivation (Fig. 1E–G). Amyloid beta deposition (Fig. 1E) and inflammatory gene expression (TNF-α and iNOS) were not increased by OA induction (Fig. 1H–J).

### OA in the AD mouse model

**Fig. 2:**
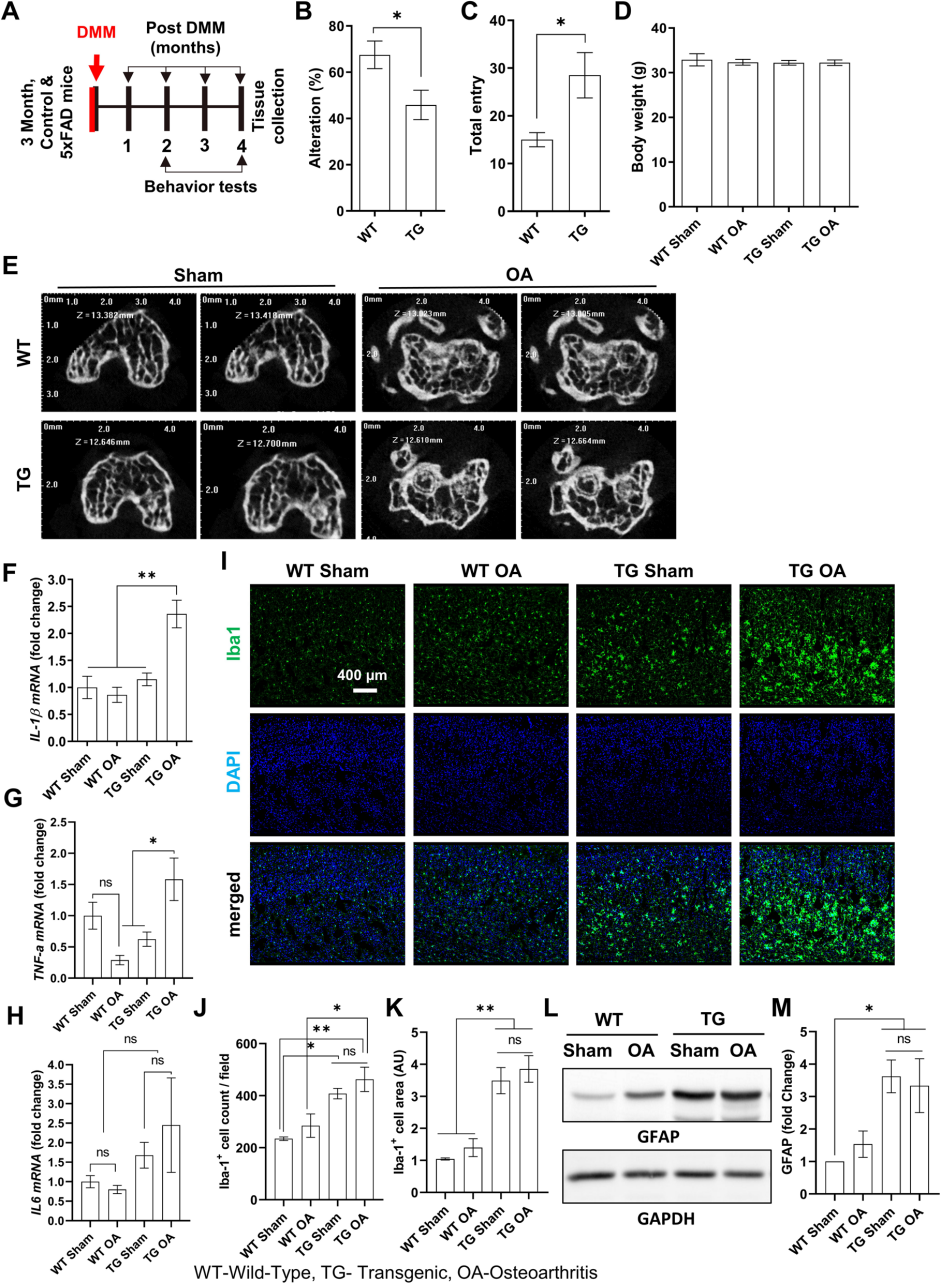
Osteoarthritis (OA) induction by surgical DMM in AD transgenic (TG) mice. **A** Timeline of surgical DMM, behavior tests, and tissue collections. Spatial working memory tests in WT and TG mice. **B** Percentage of Y-maze alterations. **C** Total number of arm entries (n = 4/group). Student’s *t*-test was used for statistical analyses. **D** Average body weight at 4 months after DMM surgery (n = 4). **E** Representative images showing the coronal view of the subchondral bone in the micro-CT scan. **F–H** Inflammatory cytokine (IL-1β, TNF-α, and IL-6) mRNA expression in brain tissues was measured using real-time PCR. **I** Representative images showing Iba-1 immunofluorescence staining in sham and surgical DMM-induced mouse brain tissues obtained from WT and TG mice. **J** Iba-1-positive cell counts. **K** Area of Iba-1-positive cells in TG mice (at least 16 images/group). **L** GFAP protein expression in brain tissues was measured using Western blot analysis. Band intensities were normalized to GAPDH. **M** Fold changes in GFAP expression per WT sham control. Values are presented as mean ± SEM (n = 4/group). One-way ANOVA with Tuckey test was used. ***P < 0.001 **P < 0.01 *P < 0.05 were considered as significant for comparisons; *ns* not significant

The 5xFAD transgenic (TG) mice, which carry five different human AD mutations, were used in our study. AD pathologies, including neuroinflammation, amyloid plaque deposition, and cognitive deficits, clearly appeared after 6 months of age (Fig. 2B and C). Therefore, surgical DMM was performed on 3-month-old mice to induce OA before AD pathology (Fig. 2A, scheme). Brain and knee tissues were collected 4 months after surgery. Micro-CT scan images of the knee confirmed OA induction both in WT and TG mice, indicating severe bone remodeling, such as cartilage surface fibrillation and proteoglycan (Fig. 2E). The left-brain hemisphere was used for histological analyses, whereas the right-brain hemisphere was used for biochemical analyses, such as RT-PCR, and Western blotting. The mRNAs for inflammatory cytokines IL-1β and TNF-α were significantly upregulated in the TG OA group vs. the TG sham group (Fig. 2F–H). Sagittal sections of the hemispheres were immunostained using an anti-Iba-1 antibody. The number and size of microglia were dramatically increased in the brain tissues of TG mice compared with those of WT mice (Fig. 2I, J, and K). The expression level of an astrocytic marker, the GFAP protein, in TG mice was significantly upregulated, whereas OA induction did not affect the GFAP level (Fig. 2L and M).

### OA induction increased amyloid beta deposition in AD mice

**Fig. 3:**
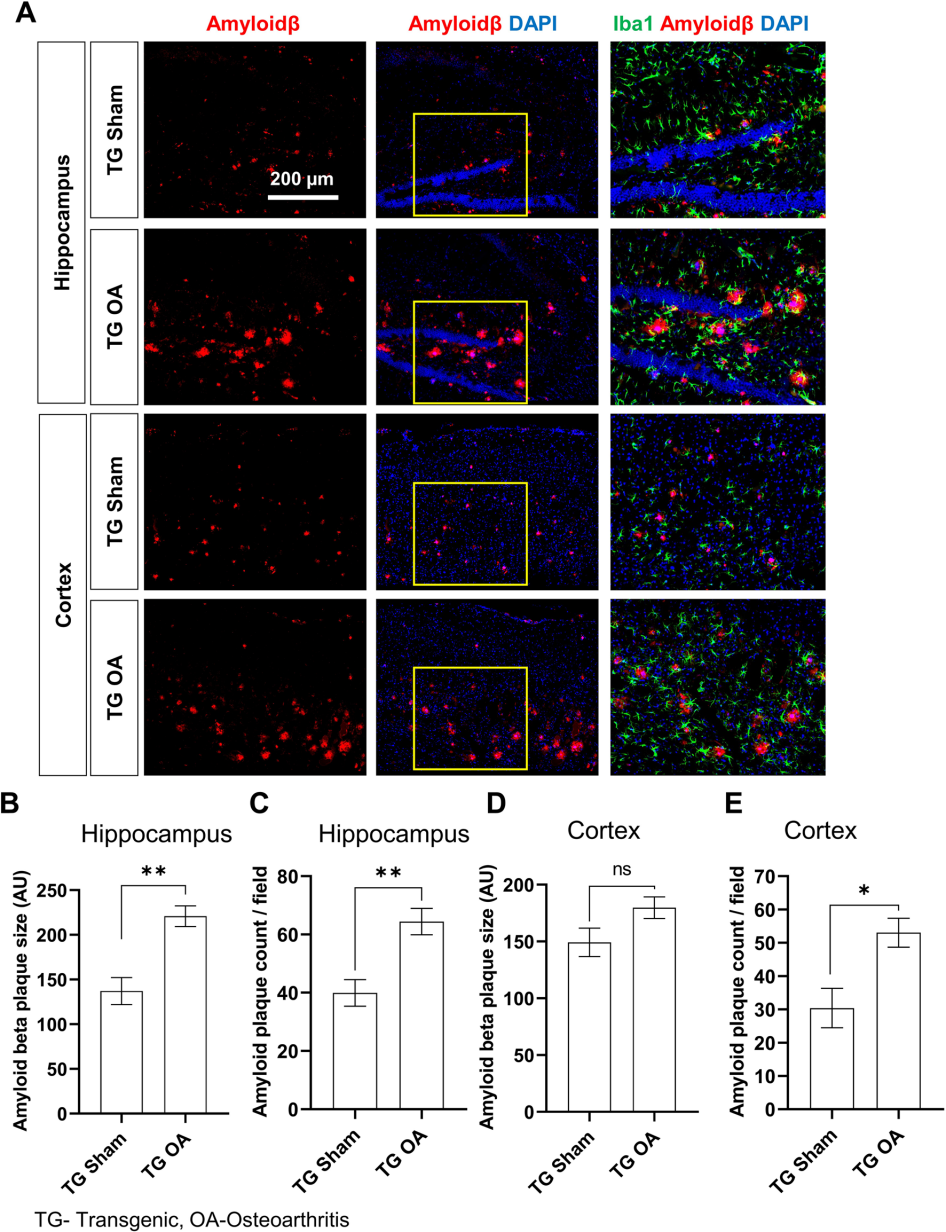
Amyloid beta deposition in the brain tissues of 5xFAD TG mice with or without OA. **A** Representative immunofluorescence images showing amyloid plaques in the hippocampus and cortex of TG mouse brains 4 months after surgical DMM. Graphs showing **B, D** amyloid beta plaque size and **C, E** amyloid beta plaque count per field in the hippocampus and cortex of TG sham and TG OA mice (at least 16 brain images/group). Values are expressed as mean ± SEM (n = 4/group). Student’s *t*-test was used for statistical analyses. **P < 0.01, *P < 0.05 were considered significant for the comparison between TG sham and TG OA; *ns* not significant

AD is characterized by amyloid beta plaques and neuronal degeneration in the brain [14]. Amyloid beta accumulation in the hippocampus and cortical areas was measured using immunohistochemistry. Numerous amyloid plaques were observed in the brains of TG mice (Fig. 3A). OA induction in TG mice increased the average size of amyloid plaques in the hippocampus and the cortex compared with TG sham mice (Fig. 3B and D). The number of amyloid plaques was also higher in both brain regions of TG OA mice compared with TG sham mice (Fig. 3C and E).

### OA induction potentiated neuronal loss in the AD mouse model

**Fig. 4:**
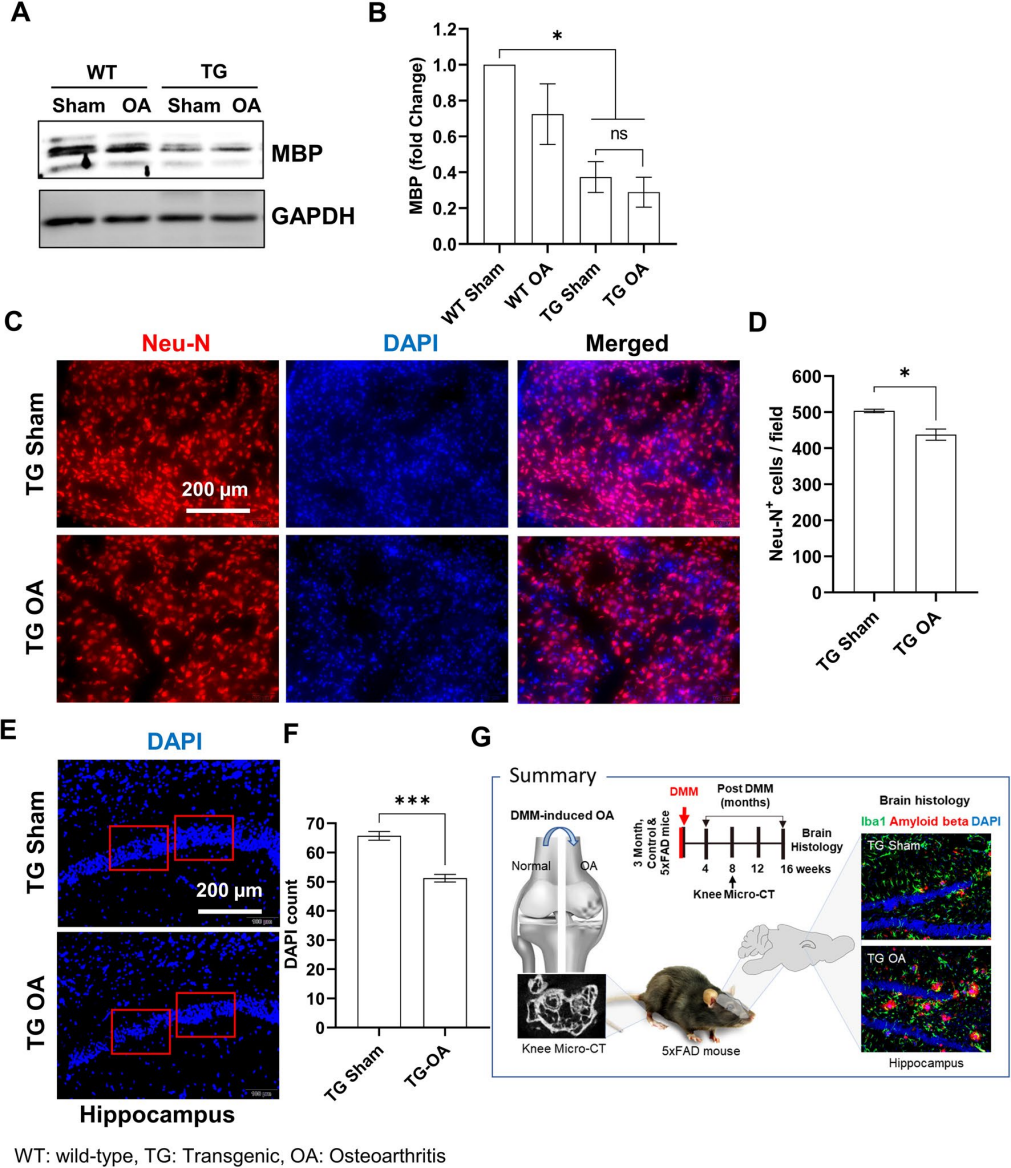
Neuronal loss in the brain tissues of TG mice with or without OA. **A** Representative immunoblot of MBP protein expression in brain tissues of TG mice with or without OA. **B** MBP protein fold change compared with the WT sham control. Band intensities were normalized to GAPDH (n = 4/group). One-way ANOVA with Tuckey test. **C** Representative images showing Neu-N immunostaining in the cortex region of mice brains 4 months after surgical DMM. **D** Neu-N-positive cell count per field. **E** Representative images showing neurons in the hippocampal CA1 region at 4 months after surgical DMM. **F** DAPI count per field in TG sham and TG OA brain tissues (9 images per brain, n = 4). **G** Graphical summary. Values are expressed as mean ± SEM. Student’s *t*-test was used for statistical analyses. ***P < 0.001, *P < 0.05 for the comparison between TG sham and TG OA; *ns* not significant

Neuronal loss and neurodegeneration in 5XFAD mice have been reported by showing the reduction in myelin proteins and the number of neurons of hippocampus [15]. To examine neurodegeneration, the myelin basic protein (MBP) was analyzed in the brains of TG mice with induced OA. The levels of the MBP protein were significantly lower in TG mice compared with WT mice; however, induction of OA did not reduce the levels of the MBP protein (Fig. 4A and B). Furthermore, the neuronal loss was analyzed by immunostaining using an antibody for Neu-N, a neuronal marker [16]. The number of Neu-N-positive cells was significantly lower in the cortex region of the TG OA mice brain compared with that of the TG sham mice (Fig. 4C and D). TG mice with induced OA showed a reduced number of neurons in the CA1 region of the hippocampus compared with TG sham mice (Fig. 4E and F).

## Discussion

Although many researchers have been attempting to understand the mechanism of AD pathology and develop new treatments for AD, there is no drug that can cure AD. AD may be caused by a combination of many different risk factors [17, 18]. Therefore, identifying risk factors that accelerate the progression of AD will ultimately be a strategy for overcoming AD. Osteoarthritis has been thought to be a risk factor for dementia; however, in fact, several clinical studies have not found a correlation with AD [5, 19]. Perhaps this is because most patients with arthritis take anti-inflammatory drugs for a long time. In addition, because both OA and AD are geriatric diseases, it was difficult to study the two diseases separately. Therefore, we had no choice but to use an animal model to investigate the direct causal relationship between the two diseases. In this study, AD progression was observed in mice with and without knee OA at early stages of dementia; moreover, amyloid accumulation and neurodegeneration progressed faster in OA mice (graphical summary in Fig. 4G).

Neurodegenerative diseases are progressive degenerative disorders that include AD, Parkinson’s disease, multiple sclerosis, amyotrophic lateral sclerosis, and Huntington’s disease, and their incidence increases with age [20]. Among the common pathogenic mechanisms of these neurodegenerative diseases, the first is the accumulation of toxic proteins, such as amyloid beta or syn-nuclein, and the second is an increase in neuroinflammation [21]. In the brains of patients with dementia, microglia, which are cells that are mainly responsible for brain immunity, are increased and exist in an excessively activated form [22]. In particular, microglia around amyloid plaques exist in a form in which the ability to remove toxic substances and secrete regenerating substances is lost [23]. Dysfunction of microglia and increased secretion of neurotoxic factors, such as excessive inflammatory cytokines, reactive oxygen species, and NO, are commonly observed in the AD brain, leading to neuronal cell death and synapse loss, consequently accelerating the progression of neurodegenerative diseases.

OA, unlike rheumatoid arthritis, is first accompanied by mechanical degeneration of articular cartilage and structural changes in the joint, including the synovial membrane, meniscus, and periarticular, consequently causing inflammatory joint disease [24, 25]. Inflammation aggravates arthritis progression and induces arthritis in other joints. Studies of patients and animal models with OA have shown that innate inflammatory pathways triggered by pattern recognition receptor signaling are highly activated in the joint [26]. Inflammatory markers in both synovial fluid and serum are highly correlated with the progression of joint space stenosis and pain severity in osteoarthritis of the knee. In this study, the mechanism underlying how OA accelerates brain degeneration was not elucidated. Changes in inflammatory substances in the blood or synovial fluid can be predicted to act on the brain; however, further studies are necessary to elucidate the underlying molecular mechanism.

AD is a multifactorial late-onset disease. Despite the importance of genetic factors, other clinical conditions are associated with AD. Diabetes, hyperlipidemia, hypertension, infection, and brain injury are well-known risk factors for AD. Therefore, early diagnosis and treatment of these diseases are critical for delaying AD progression. In this study, we demonstrated that knee OA can be a risk factor for AD progression. Inflammatory cytokines released from inflamed knee areas can induce low levels of inflammation, leading to systemic chronic inflammation, which can trigger neuroinflammation, a major pathophysiology of AD. Kyrkanides et al. reported that localized peripheral inflammation in the knee contributes to neuroinflammation. In this report, inducible IL-1β overexpression in the knee was used as an OA model [27]. Induction of OA by producing an inflammatory cytokine showed exacerbated and accelerated amyloid plaque accumulation in the APP AD mouse brain [27].

In our study, OA was induced by transecting the medial meniscotibial ligament, causing joint destabilization. This led to a slow and gradual loss of articular cartilage, which resembled human OA conditions. Several possible mechanisms can explain how knee OA can exacerbate neuroinflammation. OA was associated with significantly higher levels of the blood proinflammatory cytokines IL-1β, IL-6, and C-reactive protein (CRP) [28]. Blood CRP levels reflect local joint inflammation in patients with advanced OA [29], and IL-1β and CRP are significant risk factors for the development of AD [30, 31]. IL-1β is also known to drive the production of β-amyloid precursor proteins, thus regulating amyloid plaque deposition in the AD brain. Furthermore, IL-1β and TNF-α can cross the blood–brain barrier [32], subsequently inducing microglial activation and leading to neuroinflammation and neurodegeneration. Further studies are needed to determine which of the substances that are secreted from the synovial fluid directly induce neuroinflammation, to elucidate the direct molecular mechanisms underlying the association between OA and AD.

Interestingly, OA did not induce neuroinflammation in WT control mice, whereas it induced neuroinflammation and accelerated amyloid beta deposition in TG mice. An increase in BBB leakage was observed in 5xFAD mice compared with WT mice, which could be a possible explanation for this phenomenon. Kook et al*.* have shown that amyloid beta disrupts tight junctions in brain capillaries, leading to BBB dysfunction in 5xFAD mice [33].

A growing number of studies have demonstrated the effectiveness of exercise and physical activity in the management of AD [34, 35]. The total distance traveled by mice with DMM-induced OA was shorter than that of sham mice. Therefore, we cannot rule out that the AD pathophysiology was exacerbated by the decreased physical activity detected in OA mice. A well-established exercise program for patients with OA and AD would be ideal as a preventive intervention.

In conclusion, OA is a risk factor for AD. Developing therapeutic agents and establishing prevention strategies should be considered according to the causes of AD. It is necessary to develop programs, including exercise, anti-inflammatory therapy, and long-term follow-up, to prevent OA-induced AD progression.

## Data Availability

Data are available upon reasonable request to the corresponding author.

## Abbreviations

OA: Osteoarthritis
AD: Alzheimer’s disease
DMM: Destabilization of medial meniscus
WT: Wild-type
NO: Nitric oxide
BBB: Blood–brain barrier
TG: Transgenic
PCR: Polymerase chain reaction
PBST: Phosphate buffer saline with triton-X 100
Iba-1: Ionized calcium binding adaptor molecule 1
GAPDH: Glyceraldehyde 3-phosphate dehydrogenase
MBP: Myelin basic protein
CRP: C-reaction protein
